# Population Analysis of O26 Shiga Toxin-Producing *Escherichia coli* Causing Hemolytic Uremic Syndrome in Italy, 1989–2020, Through Whole Genome Sequencing

**DOI:** 10.3389/fcimb.2022.842508

**Published:** 2022-02-09

**Authors:** Valeria Michelacci, Margherita Montalbano Di Filippo, Federica Gigliucci, Silvia Arancia, Paola Chiani, Fabio Minelli, Nancy H. C. Roosens, Sigrid C. J. De Keersmaecker, Bert Bogaerts, Kevin Vanneste, Stefano Morabito

**Affiliations:** ^1^ Department of Food Safety, Nutrition and Veterinary Public Health, Istituto Superiore di Sanità, Rome, Italy; ^2^ Sciensano, Biological Health Risks, Transversal Activities in Applied Genomics, Brussels, Belgium

**Keywords:** STEC, virulence plasmids, genomics, evolution, population study

## Abstract

Shiga toxin-producing *Escherichia coli* (STEC) belonging to the O26 serogroup represent an important cause of Hemolitic Uremic Syndrome (HUS) in children worldwide. The localization of STEC virulence genes on mobile genetic elements allowed the emergence of clones showing different assets of this accessory genomic fraction. A novel O26 STEC clone belonging to Sequence Type (ST) 29 and harboring *stx2a*, *ehxA* and *etpD* plasmid-borne genes has emerged and spread in Europe since the mid-1990s, while another ST29 clone positive for *stx2d* and lacking plasmid-borne virulence genes was recently described as emerging in France. In Italy, O26 has been the most frequently detected STEC serogroup from HUS cases since the late 1990s. In this study we describe the genomic characterization and population structure of 144 O26 STEC strains isolated from human sources in Italy in the period 1989-2020. A total of 89 strains belonged to ST21, 52 to ST29, two to ST396 and one to ST4944. ST29 strains started to be isolated from 1999. 24 strains were shown to harbour *stx1a*, alone (n=20) or in combination with *stx2a* (n=4). The majority of the strains (n=118) harbored *stx2a* genes only and the two ST396 strains harbored *stx2d*. A Hierarchical Clustering on Principal Components (HCPC) analysis, based on the detection of accessory virulence genes, antimicrobial resistance (AMR) genes and plasmid replicons, classified the strains in seven clusters identified with numbers from 1 to 7, containing two, 13, 39, 63, 16, 10 and one strain, respectively. The majority of the genetic features defining the clusters corresponded to plasmid-borne virulence genes, AMR genes and plasmid replicons, highlighting specific assets of plasmid-borne features associated with different clusters. Core genome Multi Locus Sequence Typing grouped ST21 and ST29 strains in three clades each, with each ST29 clade exactly corresponding to one HCPC cluster. Our results showed high conservation of either the core or the accessory genomic fraction in populations of ST29 O26 STEC, differently from what observed in ST21 strains, suggesting that a different selective pressure could drive the evolution of different populations of these pathogens possibly involving different ecological niches.

## Introduction

Shiga toxin-producing *Escherichia coli* (STEC) cause foodborne infections with symptoms ranging from mild diarrhoea to haemolytic uremic syndrome (HUS), sometimes resulting in fatal outcomes (Caprioli et al., 2005). STEC are defined as *E. coli* strains carrying the genes encoding Shiga toxins (*stx*), potent cytotoxins that block protein synthesis in target cells (Melton-Celsa, 2014), encoded by genes harboured on lysogenic bacteriophages. Two main types of *stx* genes have been described, *stx1* and *stx2*, which have been subtyped in three and eleven subtypes, respectively, so far (Scheutz et al., 2012; Lacher et al., 2016; Bai et al., 2018; Yang et al., 2019). The subtypes most often associated with severe disease and outbreaks are *stx2a*, *stx2c* and *stx2d* (Melton-Celsa, 2014; Krüger and Lucchesi, 2015).

The ability of STEC to cause severe disease is also associated with the acquisition of accessory virulence genes carried by mobile genetic elements, such as pathogenicity islands (PAIs) and plasmids, mainly involved in bacterial adhesion to the intestinal mucosa of the host. The “attaching and effacing” adhesion mechanism, encoded by the PAI termed Locus for Enterocyte Effacement (LEE), is a typical feature of certain STEC, including those belonging to the “top-5” serogroups O157, O26, O103, O145 and O111, which are those most frequently reported as causative agents of severe cases of infections and outbreaks worldwide. Other PAIs associated with the “top-5” serogroups are the OI-122, carrying genes whose products are involved in favouring the colonization of the host, such as *efa1/lifA*, and the OI-57, carrying *adfO* gene, promoting adherence to the host cells. STEC strains most often associated with severe disease in humans often possess large virulence plasmids, such as pO157 described in O157 strains and similar plasmids described in STEC of other “top-5” serogroups, which harbor additional virulence genes including *ehxA*, *katP*, *espP*, *etpD* and *toxB*, encoding an enterohemolysin, a catalase peroxidase, a serine protease, an adherence factor and a type II secretion system effector, respectively (Caprioli et al., 2005; Fratamico et al., 2011).

Historically, O157 has been the most frequently reported STEC serogroup in association with severe disease worldwide (Caprioli et al., 2005), but the number of severe cases associated with O26 STEC has been steadily increasing in the last decade in several countries and particularly in Europe, where it was associated with 20.1% of confirmed cases of human STEC infections in 2020 (EFSA and ECDC, 2021). Cattle is an important natural reservoir for O26 STEC and the transmission route to humans is mainly represented by the ingestion of contaminated food or contact with wild animals, but also person to person transmission has been reported (Brown et al., 2012; Scavia et al., 2018).

The vast majority of O26 STEC belongs to two major sequence types (STs), ST21 and ST29, both comprised in Clonal Complex 29. While the presence of *stx2* genes has been extensively reported in both STs, *stx1* genes have been associated almost exclusively to strains belonging to ST21, alone or in combination with *stx2* (Bielaszewska et al., 2013; Ishijima et al., 2017). The *stx1* subtype associated with O26 STEC is *stx1a*, while *stx2a* represents the main *stx2* subtype, with strains harbouring *stx2b* (Smith et al., 2017), *stx2d* (Delannoy et al., 2015) and *stx2f* (Grande et al., 2016) also sporadically described.

A highly virulent O26 STEC clone, belonging to ST29 and harbouring *stx2a*, emerged in Germany in the mid-1990s and spread rapidly throughout Europe (Bielaszewska et al., 2013). This novel clone harbours a reduced combination of the plasmid-borne virulence genes *ehxA*, *katP*, *espP* and *etpD.* More specifically, this STEC O26 ST29 *stx2a* clone only possesses the *ehxA* and *etpD* genes. Interestingly, the emergence of other ST29 O26 STEC clones was reported also in other countries in recent years, each associated with a different repertoire of these virulence genes. Among these, a clone harbouring *stx2d* and lacking all the four plasmidic genes was described in France (Delannoy et al., 2015), while another clone, described in Japan, showed the presence of *stx2a* and the plasmidic genes arrangement *ehxA*+, *katP*-, *espP*+, *etpD*-.

HUS is considered a sentinel event revealing the circulation of STEC in the general population (Loconsole et al., 2020). In Italy, the surveillance of HUS cases started in the late 1980s and showed that O26 has been the most frequently detected STEC serogroup among HUS cases in the Italian pediatric population since the late 1990s (Scavia et al., 2018). Moreover, this serogroup has been involved in large outbreaks in recent years in Italy (Germinario et al., 2016; Scavia et al., 2018).

In this study, we describe O26 STEC strains isolated from human cases of disease that occurred in Italy during a period of thirty years. We report on the genomics characterization of the O26 STEC clones causing severe disease in Italy and investigated the population structure through Hierarchical Clustering on Principal Components (HCPC) and phylogenomics analysis based on the core genome of the isolates.

## Materials and Methods

### Bacterial Strains and Whole Genome Sequencing

In this study, we analyzed a total of 144 O26 STEC strains isolated from human sources in Italy in the period 1989-2020 by the National Reference Laboratory for *E. coli* at the Istituto Superiore di Sanità as part of the national surveillance program for HUS. In detail, 53.5% of the strains (n = 77) were isolated from cases of HUS, 7.6% from bloody diarrhea (n = 11) and 4.9% from cases of diarrhea (n = 7). The remaining strains were isolated from patients for which the symptoms were unknown (24.3%, n = 35) and from contacts of cases of disease (9.7%, n = 14). This collection of strains represented 20.3% of all the STEC isolated in Italy from human cases of disease in the same period (144/708) and in detail included 30.2% of all the STEC isolated from HUS cases (77/255).

To perform whole genome sequencing (WGS), from a 2 mL overnight TSB culture of each strain grown at 37°C, total DNA was extracted with the GRS Genomic DNA Kit Bacteria (GRISP Research Solutions, Porto, Portugal). The majority of the sequences were produced with Ion Torrent sequencing technology (Thermo Fisher Scientific, MA, USA) by preparing sequencing libraries of about 400 bp from 100 ng of total DNA using the NEBNext Fast DNA Fragmentation & Library Prep Set for Ion Torrent (New England BioLabs, MA, USA), then treated through emulsion PCR and enrichment on the Ion OneTouch 2 System and finally sequenced on an Ion Torrent S5 platform (Thermo Fisher Scientific, MA, USA) using the ION 520/530 KIT-OT2 (Thermo Fisher Scientific, MA, USA) according to the manufacturer’s instructions. Sequencing of some samples was instead outsourced and performed on an Illumina HiSeq 2000 or Illumina MiSeq sequencer with a 100 bp or 250 bp paired-end protocol. Details on the sequencing technologies used for each strain are provided in **Supplementary Table 1**. All the genomic sequences are available at the European Nucleotide Archive at the European Molecular Biology Laboratory (accession no. PRJEB48948).

### Characterization of Bacterial Strains Through WGS Analysis

The sequencing reads were analyzed through the bioinformatics workflow dedicated to Shiga toxin-producing *Escherichia coli* analysis developed by Sciensano (Bogaerts et al., 2021) and made available through the Sciensano Galaxy Instance for bioinformatics analyses (https://galaxy.sciensano.be, STEC pipeline version 1.0). In brief, the workflow automatically returned quality check reports and performed assembly of reads in contigs using SPAdes v3.13 (Bankevich et al., 2012) and strain characterization in terms of serotyping (Joensen et al., 2015), virulotyping (Joensen et al., 2014), seven-genes Multi Locus Sequence Typing (MLST) (Wirth et al., 2006; Jaureguy et al., 2008), detection of plasmid replicons (Carattoli and Hasman, 2020) and antimicrobial resistance (AMR) genes. More specifically, the detection of antimicrobial resistance genes was performed by comparing and merging the results produced by the pipeline using ResFinder (Zankari et al., 2012) and ARG-ANNOT (Gupta et al., 2014) databases.

The *stx*-subtyping was performed on the Galaxy public server ARIES (Istituto Superiore di Sanità, https://www.iss.it/site/aries) (Knijn et al., 2020) through the Shiga toxin typer tool v2.0 (https://github.com/aknijn/shigatoxin-galaxy), an optimised blastn search against the sequence database of *stx* subtypes developed by the Statens Serum Institut (https://bitbucket.org/genomicepidemiology/virulencefinder_db/src/master/stx.fsa).

### Hierarchical Cluster Analysis

A multivariate data analysis was performed on the results of the characterization obtained through WGS and the descriptive data of the isolates (**Supplementary Table 1**). In particular, we conducted a preliminary Multiple Correspondence Analysis (MCA) followed by the Hierarchical Clustering on Principal Components Analysis (HCPC) methodology, using the statistical software R version 3.5.1 (http://cran.r-project.org).

We decided to employ the HCPC approach as it allows to combine the three standard methods used in multivariate data analyses (Lê et al., 2008): (i) principal component methods, (ii) hierarchical clustering, and (iii) partitioning clustering, particularly by using the k-means method. Furthermore, HCPC allows the characterization of clusters of specimens based on all the characteristics and subsets of characteristics, weighting all of them equally. The goal was to build a tree structure showing hierarchical relations between individuals or groups of individuals and detect a “natural” number of classes in the observations under study.

In detail, the dataset consisted of 83 qualitative variables: the majority were represented by genetic features, consisting out of 22 antimicrobial resistance (AMR) genes, 34 virulence genes and 27 plasmid replicons, detected in at least one of the strains tested. In addition, the detected *stx* subtype was considered as an additional single variable, including for each strain the complete profile of *stx* subtypes detected. The result of MLST, year of isolation and geographic region of isolation were also included as variables in the analysis, for a total number of 87 variables used (**Supplementary Table 1**).

Both MCA and HCPC analyses were performed using the FactoMineR package (Lê et al., 2008). The results were extracted and visualized using the Factoextra package (Kassambara and Mundt, 2020).

### Phylogenomics Based on Core Genome Multi Locus Sequence Typing

Phylogenomics analysis was performed through core genome MLST (cgMLST) using the chewBBACA tool (Silva et al., 2018) available on the Galaxy public server ARIES (Istituto Superiore di Sanità, https://www.iss.it/site/aries) (Knijn et al., 2020) using the scheme developed by the INNUENDO project, which comprises 2,360 loci in total (Llarena Ak and et al., 2018; Silva et al., 2018). The pairwise comparison was considered reliable when >80% of loci for each sample were assigned to an allele. The distances between strains were calculated by pairwise comparison of the allelic profiles using the chewTree tool available on ARIES (Knijn et al., 2020). For each pair of samples, the alleles which were not found, only partially found, or not correctly assigned to any locus were excluded from the analysis, as previously described (Gigliucci et al., 2021). The dendrogram was visualized with FigTree version 1.4.4 (https://github.com/rambaut/figtree/releases) and modified in iTOL (Letunic and Bork, 2007).

### Plasmids Comparison

The Prokka tool (Galaxy Version 1.14.5) (Seemann, 2014) was used through the Galaxy public server ARIES for the functional annotation of the assembled sequences, using the *E. coli* specific gene database and default parameters. Blast Ring Image Generator (BRIG) software v0.95 (Alikhan et al., 2011) was used with default parameters to compare the assembled genomes of O26 STEC produced in this study with the reference sequences from pO26-Vir (RefSeq accession no. NC_012487.1) and pR444_A (Cointe et al., 2018) (RefSeq accession no. NZ_QBDM01000004.1). The former is a representative virulence plasmid harbouring *ehxA*, *katP*, *espP* and *toxB* virulence genes described in O26 STEC, while the latter is a mosaic plasmid harbouring virulence genes associated with extraintestinal pathogenic *E. coli* (ExPEC) and a cassette of AMR genes (Cointe et al., 2018), recently described in O26 STEC strains isolated in Italy (Gigliucci et al., 2021). In the comparison the analyzed strains were grouped by HCPC cluster.

## Results

### Basic Genomic Characterization of O26 STEC Strains

The median number of contigs obtained in the generated assemblies was 210 and the median N50 value was 83786 bp (**Supplementary Table 1**). Among the 144 strains tested, 89 belonged to ST21 and 52 to ST29, while two strains were typed as ST396 (single locus variant of ST29) and one as ST4944 (single locus variant of ST21). All the strains isolated in the period 1989-1998 were typed as ST21, while ST29 strains appeared from 1999 onwards.

A total of 24 strains harboured *stx1* genes, subtype *stx1a*, alone (n=20) or in combination with *stx2a* (n=4). All the strains harbouring *stx1a* genes belonged to ST21, with the only exception of the strain ED0886 typed as ST4944. The vast majority of the strains harboured *stx2* genes only, typed as *stx2a* in 66 strains of ST21 and 52 strains of ST29, and as *stx2d* in the two strains of ST396.

All the strains displayed the presence of the virulence genes *eae*, *espA*, *espB* and *tir*, all encoded on the LEE locus, as well as *nleB*, a non-LEE encoded effector of the Type Three Secretion System. As for antimicrobial resistance genes, all the tested strains were positive for the presence of (*Bla*)AmpC2_Ecoli, and (*Bla*)ampH genes, encoding Class C β-lactamases, and (*Bla*)Penicillin_Binding_Protein_Ecoli, encoding a modulator of β-lactam resistance.

### Hierarchical Cluster Analysis

Multiple Correspondence Analysis showed that >50% of the variance observed in the dataset could be ascribed to seven dimensions.

**Figure 1:**
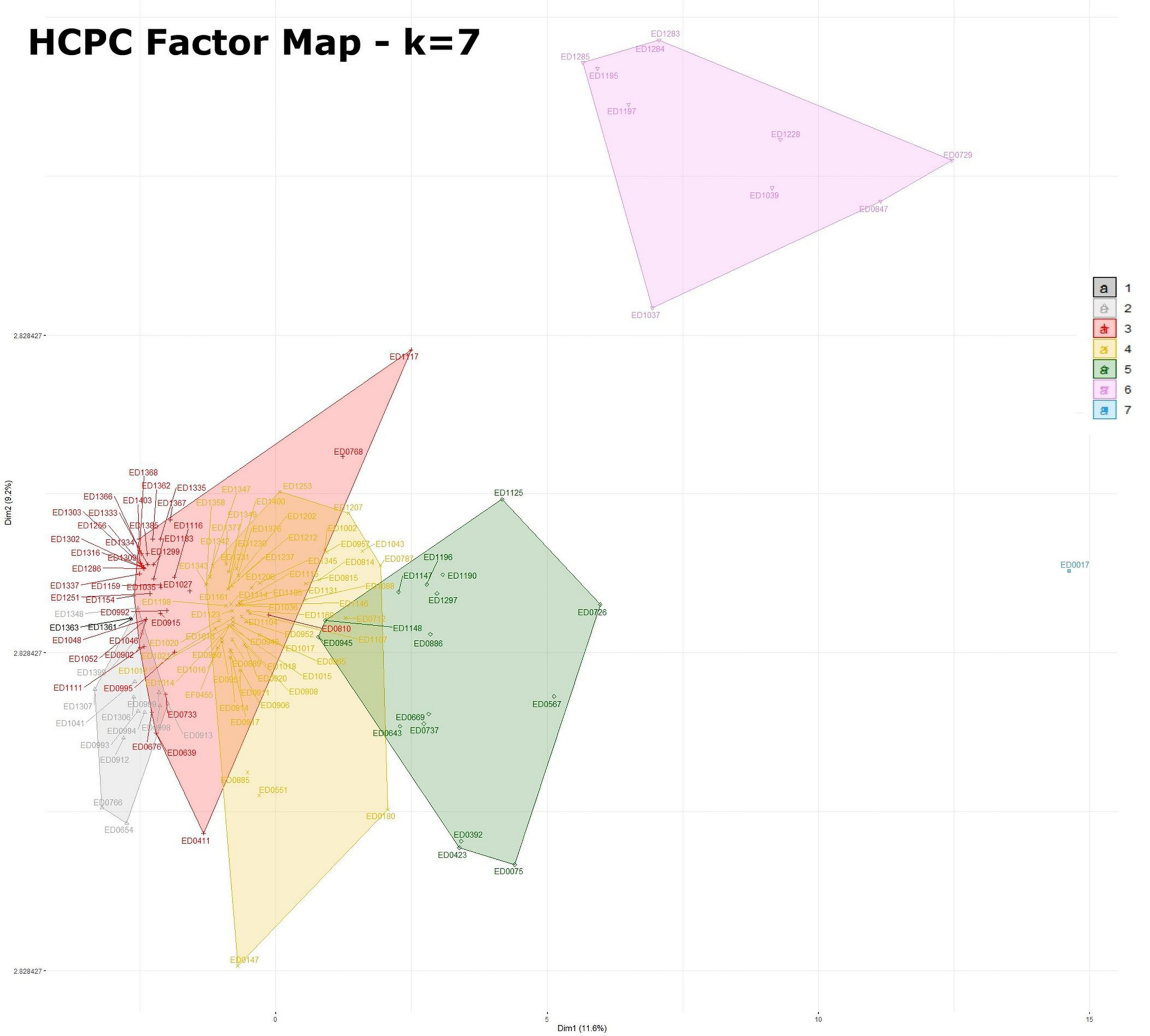
Factor map obtained by HCPC analysis with 7 dimensions. The legend for the colors indicating the clusters is included in the figure. Statistics for HCPC analysis are reported in **Supplementary Table 2**.

**Figure 2:**
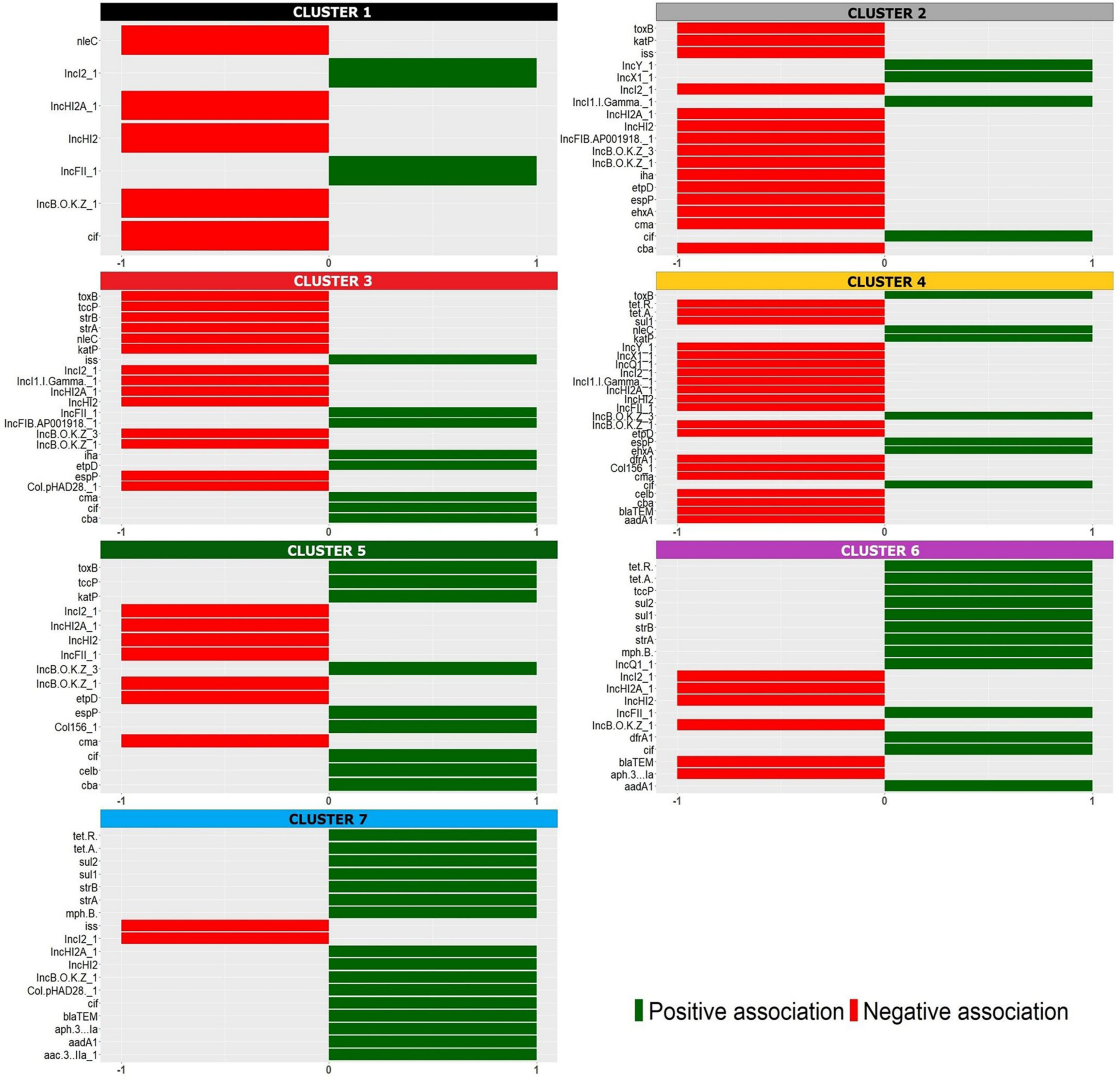
Positive (green) and negative (red) association of the variables with the identified HCPC clusters, identified with the colors used in the factor map (**Figure 1**).

Seven well-defined clusters (k = 7) were generated with the HCPC analysis, (**Figure 1**). The significant positive (presence, reported as 1) and negative (absence, reported as 0) association of the analyzed features originating the clusters is reported in detail in **Figure 2** and in **Supplementary Table 2**. It is interesting to note that the majority of these variables corresponded to virulence genes typically harboured on STEC virulence plasmids (Caprioli et al., 2005; Fratamico et al., 2011), antimicrobial resistance genes often carried by plasmids (Cointe et al., 2018) and plasmid replicons.

The seven clusters identified (**Figure 1** and **Supplementary Table 2**) were composed as follows. Cluster 1 grouped two isolates [1.4% (2/144)] typed as ST29 and was characterized by the presence of different plasmid replicons (IncI2_1 and IncFII_1). Cluster 2 was composed by 13 isolates [9% (13/144)], 12 of which typed as ST29 and only one as ST21, and was defined by a negative association with the main plasmid-borne virulence genes *ehxA*, *katP*, *espP*, *etpD* and *toxB*. Cluster 3 contained 39 strains [27% (39/144)], 38 of which typed as ST29 and one as ST21, and was positively associated with the presence of *etpD*, *cba* and *cma* plasmid-borne virulence genes. Cluster 4 was composed of 63 strains [43.75% (63/144)], including 61 strains belonging to ST21 and two strains typed as ST396, and was characterized by a positive association with the plasmid-borne virulence genes *ehxA*, *katP*, *espP*, and *toxB.* Cluster 5 included 16 isolates [11.1% (16/144)], 15 out of which belonging to ST21 and one to ST4944, and was described by a positive association, amongst others, with *toxB*, *katP* and *espP* plasmid-borne genes and a negative association with *etpD*. Cluster 6 consisted of 10 strains [6.9% (10/144)] all belonging to ST21 and presenting the antimicrobial resistance genes *dfrA1, aadA1, sul1, sul2, tetA, tetR, strA* and *strB* together with IncFIC_1, IncFII_1 and other plasmid replicons. Finally, cluster 7 only included one ST21 isolate having the oldest isolation date in the collection (ED0017 from the year 1989) and was positively associated with a high number of variables (**Figure 2** and **Supplementary Table 2**) mainly including AMR genes and plasmid replicons.

**Figure 3:**
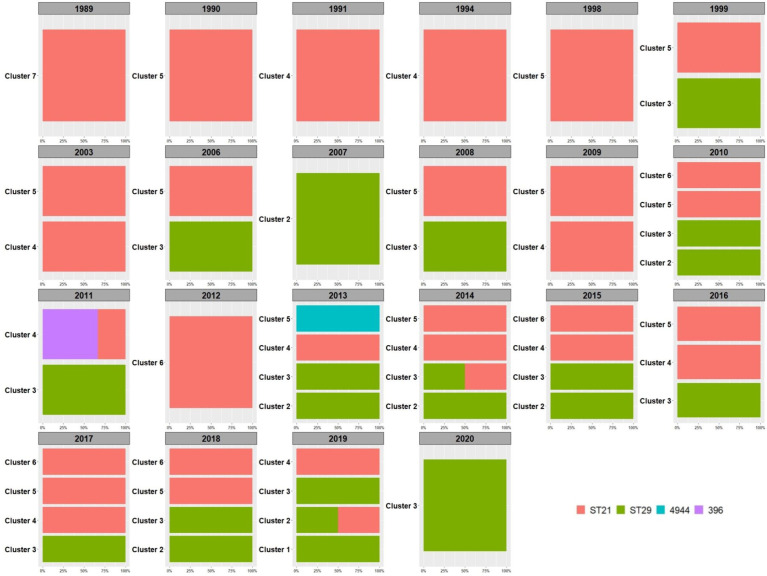
Distribution of HCPC clusters in time. The percentage of the detected Sequence Types is reported within each year and per cluster. The legend for the colors indicating the STs is included in the figure.

When inspecting the distribution of the clusters and ST types over time (**Figure 3**), ST29 belonging to cluster 3 were the first isolates of such ST to be identified in Italy in 1999, ten years after the first isolation of the first O26 STEC strain belonging to cluster 7 and typed as ST21 (**Figure 3**). Since their first isolation, ST29 strains were isolated from cases almost every year eventually representing 37% (52/139) of the strains isolated thereafter.

**Figure 4:**
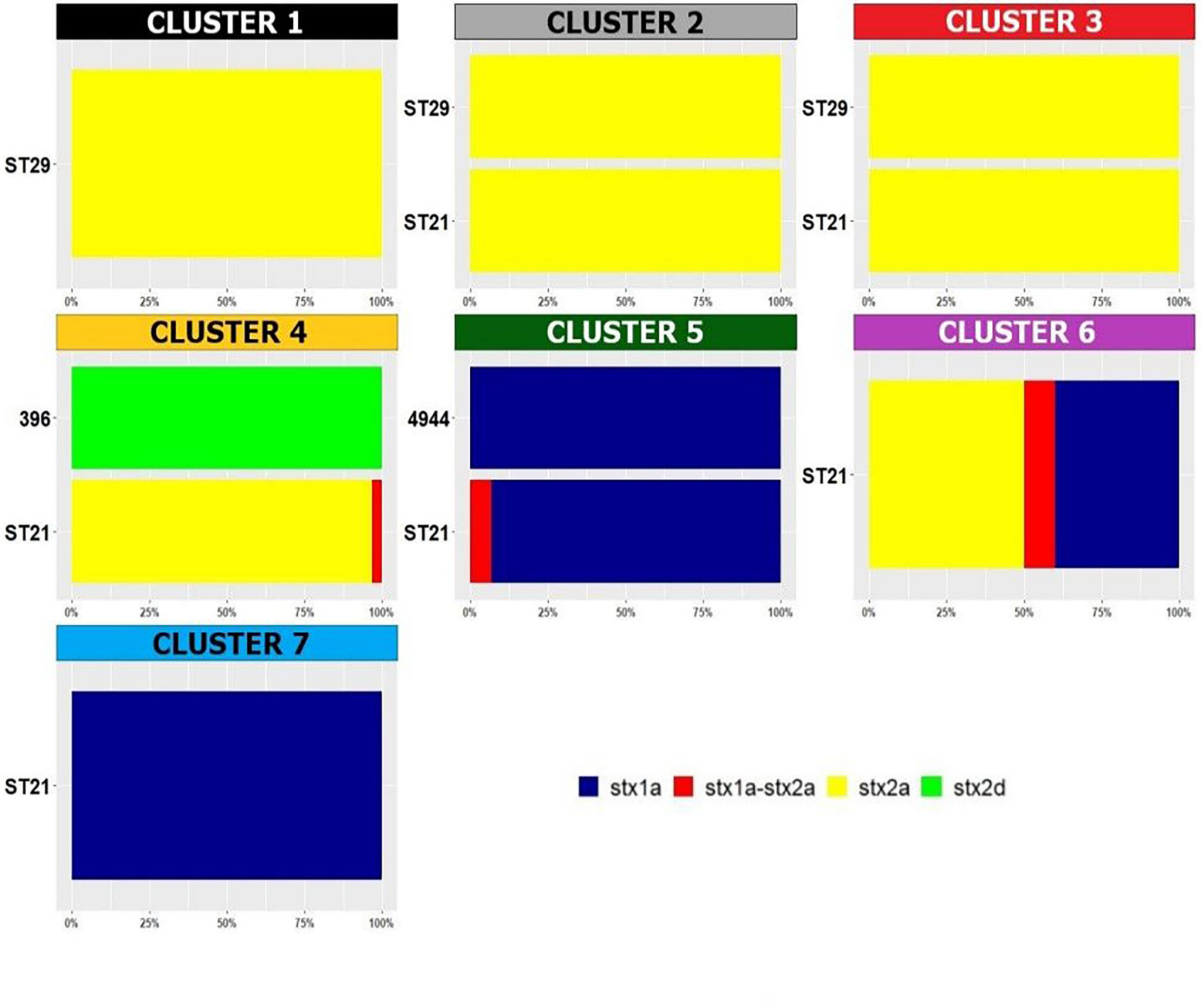
Distribution of Sequence Types in the HCPC clusters. The percentage of the detected *stx* subtypes is reported within each cluster and per each ST. The legend for the colors indicating the *stx* subtypes is included in the figure.

Regarding the detected *stx* subtypes, it’s interesting to note that the *stx* subtyping variable was positively associated with different clusters (**Supplementary Table 2** and **Figure 4**): cluster 3 showed *stx2a* only; cluster 4 mainly showed *stx2a* and also included two isolates showing the subtype *stx1a stx2a* and two isolates harbouring *stx2d*, representing the only two ST396 strains of the collection (**Figure 4** and **Supplementary Table 2**). The *stx1a* subtype was present in clusters 5, 6 and 7, which grouped mainly ST21 strains (**Figure 4**).

### Comparison of Virulence Plasmids

The results of the HCPC analysis brought the attention to different patterns of plasmid-borne virulence genes across the identified clusters. The alignment of the annotated contigs with pO26-Vir reference plasmid showed that the strains belonging to the clusters 1, 2 and 3, mainly typed as ST29, produced different patterns of alignment. Genomes of strains from clusters 1 and 2 produced the largest alignment with the reference sequence in terms of total length of aligned regions, while the sequences of the strains grouped in cluster 3 covered the smallest area of the pO26-Vir sequence (**Supplementary Presentation 1**). More specifically, 79.5% (31/39) of the strains of cluster 3 showed sequence identity in the region including the locus harbouring *ehxA* gene (12583-15579 bp), while only six of the strains (15.4%) produced sequences covering with high sequence identity almost the whole length of the reference plasmid. Finally, two strains apparently lacked the whole plasmid (**Supplementary Presentation 1**). It is interesting to note that clusters 1, 2 and 3 lacked the *katP* gene (164088-166298 bp), while the *toxB* gene (39589-49089 bp) was present in the two strains of cluster 1 and completely absent in the strains from clusters 2 and 3. Among these three clusters, all containing mostly ST29 strains, the *espP* gene (57774-61676 bp) was only detected in both the strains assigned to cluster 1 and 30.7% of the strains in cluster 2 (4/13), while it was absent in all the isolates grouped in cluster 3 (**Supplementary Presentation 1**).

When considering the strains belonging to the clusters 4, 5, 6 and 7, including all but two ST21 strains, the alignment showed that they shared the vast majority of the plasmid structure with the reference sequence. Nevertheless, the region encoding translocation elements for conjugative transfer (*tra* genes, 109940-134889 bp) was present only in a few strains, including 9.5% of the strains of cluster 4 (6/63), 18.8% of cluster 5 (3/16) and 10% of cluster 6 (1/10). The complete conjugative region was instead detected in the only strain forming cluster 7, which was positive for the *ehxA* and *katP* genes, but negative for *toxB* and *espP* genes (**Supplementary Presentation 1**).

A few ST21 strains lacked the whole pO26-Vir plasmid: five strains of cluster 4, one of cluster 5 and two of cluster 6 (**Supplementary Presentation 1**).

Based on the results of HCPC analysis, it was also possible to identify in clusters 6 and 7 a pattern of features typical of the mosaic plasmid pR444_A, harbouring virulence genes associated with extraintestinal pathogenic *E. coli* (ExPEC) and a cassette of AMR genes (Cointe et al., 2018), described in STEC strains belonging to the O80 serogroup and recently also found in O26 STEC strains isolated in Italy (Gigliucci et al., 2021). The annotated contigs of the strains investigated were thus aligned also to the reference sequence of pR444_A. This analysis displayed the presence of sequences aligning on the reference in 60% (6/10) of the strains composing cluster 6, including the two strains (ED1283 and ED1284) previously described to carry this plasmid (**Supplementary Presentation 2**) (Gigliucci et al., 2021). More specifically, 50% (5/10) of the strains in cluster 6 possessed most of the features identified in pR444_A, including *iroN* (20413-22590 bp), *sitABCD* (154242-157691 bp), *iucABCD* (145222-150910 bp) and *hlyF* (164326-165435 bp) virulence genes and an extended AMR region (40881-86037 bp). These isolates only lacked the *etsABC* locus encoding a putative Type I Secretion System, present in the reference sequence of the pR444_A plasmid. One strain (ED1228) also lacked *hlyF* and *iroN* virulence genes and *bla* genes from the AMR region. The remaining four strains harboured an incomplete conjugative *tra* region (92765-122839 bp) similar to that present in the reference pR444_A plasmid and fragments of the AMR region and all but one (ED0729) carried the *iucABCD* locus. The alignment also showed sequences matching the reference pR444_A plasmid in the strain classified in cluster 7 (**Supplementary Presentation 2**).

The strains of the clusters 1, 2, 3, 4 and 5 did not show the presence of plasmids similar to the pR444_A (**Supplementary Presentation 2**), except for a putative *iucABCD* stretch that was identified in one out of 13 strains of cluster 2, in 74.4% (29/39) of cluster 3, 52.4% (33/63) of cluster 4 and 93.8% (15/16) of cluster 5 (**Supplementary Presentation 2**).

### Phylogenomics Investigation

**Figure 5:**
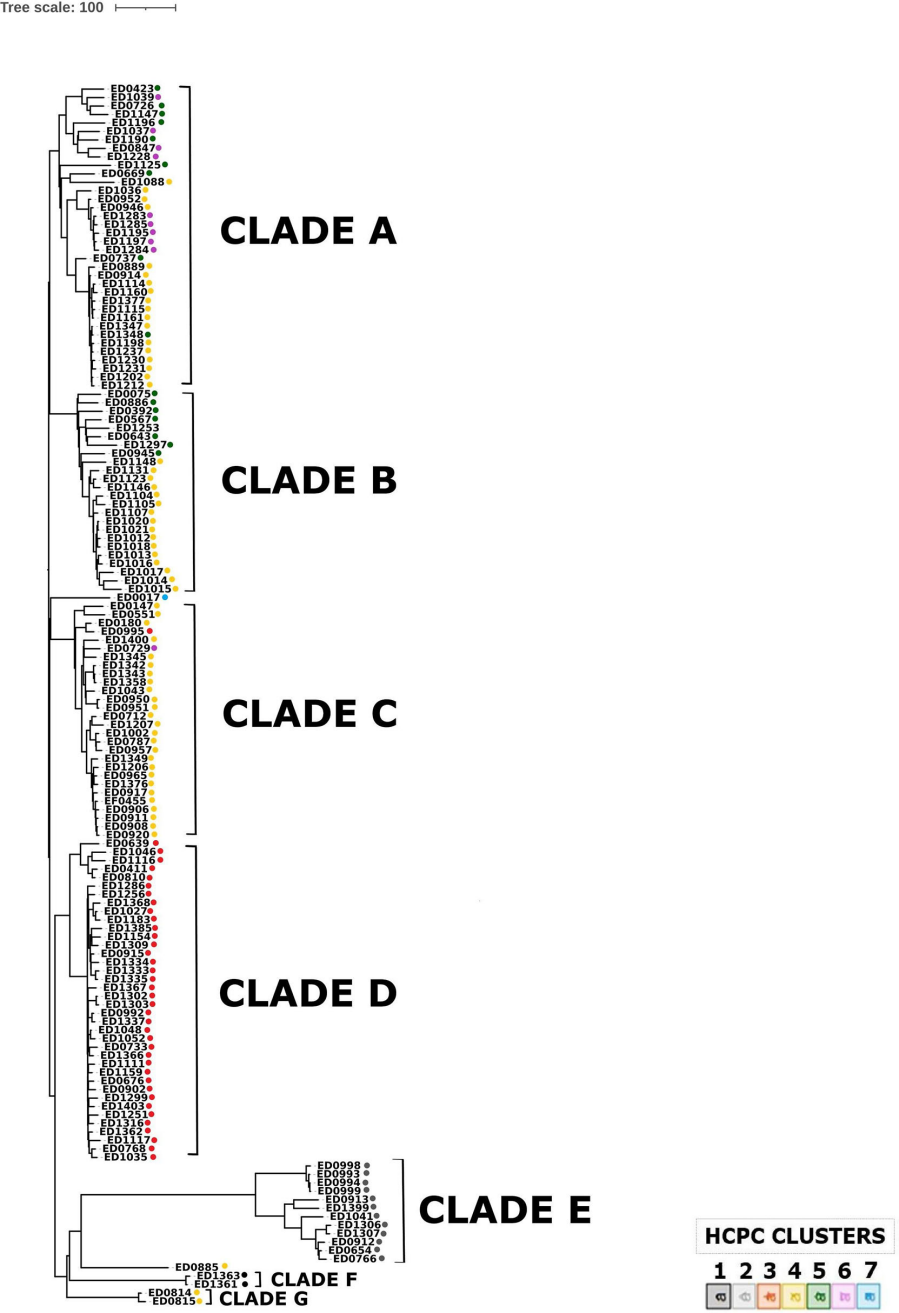
Phylogenomic analysis of O26 STEC strains through cgMLST. The colors indicate the clusters attributed to each strain by HCPC analysis.

In order to understand the evolution of O26 STEC causing disease in Italy, a phylogenetic analysis was performed based on cgMLST. The resulting dendrogram is shown in **Figure 5**. The analysis grouped ST21 strains in three main clades (A-C). The ED0886 strain, the only representative of ST4944 in this collection, was part of Clade B together with ST21 strains. On the other hand, ST29 strains were divided into two main clades (D and E) and an additional clade (F) grouping only the two strains ED1361 and ED1363, which correspond to the strains forming Cluster 1 in HCPC. A separate clade (G) was also composed by the only two strains belonging to ST396, ED0814 and ED0815 (**Figure 5**). The strains ED0017 (ST21) and ED0885 (ST21) represented outliers in this analysis.

The maximum number of allelic distances within clades was much lower in some clades than in others. In detail, the maximum number of allelic differences (AD) detected within the whole collection was 617, with Clade A grouped in 179 AD, Clade B in 128 AD, Clade C in 192 AD, Clade D in 112, Clade E in 227, while Clades F and G were composed of two strains each, showing eight and 15 AD, respectively. It is interesting to note that 33 out of the 38 strains grouped in Clade D differed for maximum 44 AD. The latter clade showed a low variability also in the genetic features acquired through horizontal transfer. As a matter of fact, the whole Clade D appeared homogeneous also in the HCPC analysis, entirely corresponding to Cluster 3. Similarly, the other main clade grouping ST29 strains, Clade E, corresponded to HCPC Cluster 2 and Clade F, grouping two ST29 strains, corresponded to cluster 1. On the other hand, all the ST21 strains grouped in the same clades in cgMLST (A, B and C) were dispersed in several HCPC Clusters (4, 5 and 6).

## Discussion

STEC infection is the primary cause of HUS in children worldwide. The incidence of cases associated with STEC O26 has increased considerably in Europe in the last decade. In Italy, O26 has been the most frequently detected STEC serogroup in HUS cases in the pediatric population since the late 1990s (Scavia et al., 2018).

Recent studies have highlighted the association of different combinations of virulence genes with different STEC O26 clones (Bielaszewska et al., 2013; Delannoy et al., 2015; Ishijima et al., 2017; Karnisova et al., 2018). Since the vast majority of *E. coli* virulence genes are encoded on mobile genetic elements (MGE) and can be acquired or lost through horizontal gene transfer, the association of characteristic patterns of MGE-encoded virulence genes with specific clones is interesting because it may be suggestive of different selective conditions, which could have favoured their emergence and maintenance in the ecological reservoir.

An O26 STEC clone of ST29 has been described as emerging in Europe since the mid-1990s. It harbours the *stx2a* subtype and the large virulence plasmid carrying *ehxA* and *etpD* genes but lacking the *katP* and *espP* genes (Bielaszewska et al., 2013), which were instead typically present on the pO26 virulence plasmid described in STEC O26 belonging to ST21 (Fratamico et al., 2011). On the other hand, the ST29 *stx2d*-positive clone emerging in France, completely lacked this virulence plasmid (Delannoy et al., 2015).

In this study, we used whole genome sequencing to characterize 144 STEC O26 strains isolated from human cases of disease detected in Italy by the HUS surveillance system in the last thirty years, mainly including strains isolated from severe disease such as HUS and bloody diarrhea (total 61.1%, 88/144) and covering 30.2% of all the STEC isolated from HUS cases in Italy in the period 1989-2020. The availability of such a collection prompted us to perform a population study, carried out by analyzing separately the distribution of genetic features part of the accessory fraction of the genome, including virulence determinants, AMR genes and plasmid replicons, and the core genome.

The majority (61.8%) of the analyzed strains were typed as ST21, but ST29 strains, first isolated in Italy in 1999, represented a considerable proportion (36.1%). As previously reported (Bielaszewska et al., 2013; Ishijima et al., 2017), ST29 strains were associated with the *stx2a* subtype, which was also detected in the majority (74.2%) of ST21 strains, while the *stx1a* subtype seemed to be confined to ST21 strains and to one strain belonging to ST4944, a single locus variant of ST21. No ST29 strains positive for the *stx2d* subtype, ascribable to the clone described in France (Delannoy et al., 2015), were identified. Nevertheless, two strains harbouring *stx2d* and belonging to ST396, a single locus variant of ST29, were instead observed in the Italian isolates. The higher representation of *stx2*-positive (83.3%) compared to *stx1*-positive strains (16.7%) is in line with previous knowledge about the higher association of *stx2* and particularly the *stx2a* subtype with human severe disease.

The application of HCPC analysis allowed to compartmentalize the strains in seven clusters, statistically associated with specific assets of virulence determinants, AMR genes and plasmid replicons.

It is interesting to note that the clusters grouping the ST29 strains (clusters 1, 2 and 3) in HCPC analysis are more homogeneous compared to those containing the ST21 strains (clusters 4, 5, 6 and 7) (**Figure 1**), suggesting a wider diversification of features among the latter clusters.

Among the features associated with the clusters, both positively or negatively, the virulence genes typically harboured on the pO26 virulence plasmid were the most widely represented, suggesting that different versions of the plasmid were selected and stabilized in the identified subpopulations.

The alignment of the STEC O26 sequences with pO26-Vir reference sequence confirmed the presence of a plasmid very similar to the reference in the majority of the strains belonging to clusters 4, 5, 6 and 7, including mainly ST21 strains. Interestingly, only a few of those strains harboured a complete conjugation region, which might have gone through genetic decay after stable acquisition of such plasmids in these subpopulations (**Supplementary Presentation 1**). On the other hand, the strains belonging to clusters 1, 2 and 3, including predominantly ST29 strains, showed different patterns of alignment with pO26-Vir: clusters 1 and 2 shared the majority of the sequence with the reference, with cluster 1 strains being positive for the presence of *ehxA*, *toxB* and *espP* and negative for *katP* and cluster 2 mainly lacking all these genes; cluster 3 showed instead the presence of *ehxA* locus, only (**Supplementary Presentation 1**). These results may indicate that different selective pressures could have operated on the evolution of the different clusters of strains, favouring the conservation of specific patterns of virulence genes in some of the populations identified by the different clusters. This was also manifest when considering the results of the presence of antimicrobial resistance genes. In particular, the genes *dfrA1, aadA1, sul1, sul2, tetA, tetR strA* and *strB* were positively associated with cluster 6. These genes are typically harboured on the AMR genes cassette described in the pR444_A mosaic plasmid of STEC O80 strains, harboring virulence genes typical of ExPEC such as *iucC* encoding an aerobactin, *iroN* encoding a salmochelin, *sitABCD* operon encoding an iron uptake protein, and the hemolysin *hlyF* (Cointe et al., 2018). Cluster 6 was in fact the only one containing STEC O26 strains possessing a plasmid similar to pR444_A (**Supplementary Presentation 2**). However, regions harbouring the *iucD* gene only were detected also in strains belonging to other clusters, suggesting that, in these strains, this feature could be encoded by genes present in other regions of the genome.

The cgMLST analysis showed that the clade comprising the majority (39/52) of ST29 strains (clade D) not only shared minimal allelic variation in the core genome fraction, but also completely corresponded to cluster 3 identified when analyzing the accessory genome fraction through HCPC. Moreover, also the other two clades grouping ST29 strains (clades E and F) appeared to correspond entirely to two distinct clusters identified through HCPC, with clade E corresponding to cluster 2 and clade F to cluster 1 (**Figure 5**). The low level of variation observed in the two genomic fractions, described by the clades and the clusters, identified for ST29 strains supports the hypothesis that a selective pressure might have acted in specific ecological niches during the evolution of this sequence type of STEC O26, differently from what observed for ST21 strains, which were generally much more variable. The latter group, in fact, included genomes of strains dispersed in three large clades in cgMLST analysis (clades A, B, and C), not overlapping single HCPC clusters and thus not associated with single specific patterns of accessory features. On the other hand, it has to be noted that this picture, particularly the low variability in the core genome, may also be explained by a more recent emergence of ST29, which in Italy seemed to appear only in the late 90s.

Due to the high plasticity of the *E. coli* genome the identification of conserved patterns of accessory genetic features is noteworthy. Our study showed that, since the emergence of the ST29, in the Italian STEC O26 the major evolutive drivers acted on the plasmid content, particularly by shaping the large virulence plasmid pO26-Vir and, for certain isolates, following the acquisition of a copy of the hybrid ExPEC-STEC plasmid pR444_A. These events seem to have determined the emergence of different STEC O26 populations that may have been stabilized and maintained into specific niches.

Further analyses conducted with the same approach on strains’ collections including isolates from animal, food or environmental sources, could allow getting a deeper insight into the ecology of this important STEC serogroup.

## Data Availability Statement

The datasets presented in this study can be found in online repositories. The names of the repository and accession number can be found below: https://www.ebi.ac.uk/ena, PRJEB48948.

## Author Contributions

VM coordinated the analyses, performed cgMLST analysis and drafted the manuscript, MM performed HCDC analysis, FG performed the plasmids comparisons, SA operated the analyses on Sciensano Galaxy instance, PC performed DNA extraction for sequencing, FM isolated the strains from human samples, NR, SK, BB, and KV provided the Sciensano Galaxy instance, and SM conceived the study and guided the discussion. All authors contributed to the article and approved the submitted version.

## Funding

The research that yielded the STEC WGS analysis workflow available through the Sciensano Galaxy Instance was funded by the Belgian Federal Public Service of Health, Food Chain Safety and Environment through contract RF 17/6316 StEQIDEMIC.be and by Sciensano through contract RP Be READY and RP “NGS & Bioinformatics Platform”.

## Conflict of Interest

The authors declare that the research was conducted in the absence of any commercial or financial relationships that could be construed as a potential conflict of interest.

## Publisher’s Note

All claims expressed in this article are solely those of the authors and do not necessarily represent those of their affiliated organizations, or those of the publisher, the editors and the reviewers. Any product that may be evaluated in this article, or claim that may be made by its manufacturer, is not guaranteed or endorsed by the publisher.

## Supplementary Material

Supplementary Table 1 For each tested strain the table includes: the statistics of the assembled contigs, details on sequencing technology used, region and year of isolation, and variables results used in HCPC analysis (for qualitative detection: 1 = presence; 0 = absence). The last column contains the cluster attributed to each strain through HCPC analysis.
