# Supplementary figures and images for: Population Analysis of O26 Shiga Toxin-Producing Escherichia coli Causing Hemolytic Uremic Syndrome in Italy, 1989–2020, Through Whole Genome Sequencing
Source: Front Cell Infect Microbiol. 2022 Feb 9;12:842508. doi: 10.3389/fcimb.2022.842508 (PMC8864317; doi:10.3389/fcimb.2022.842508)

## Slide 1
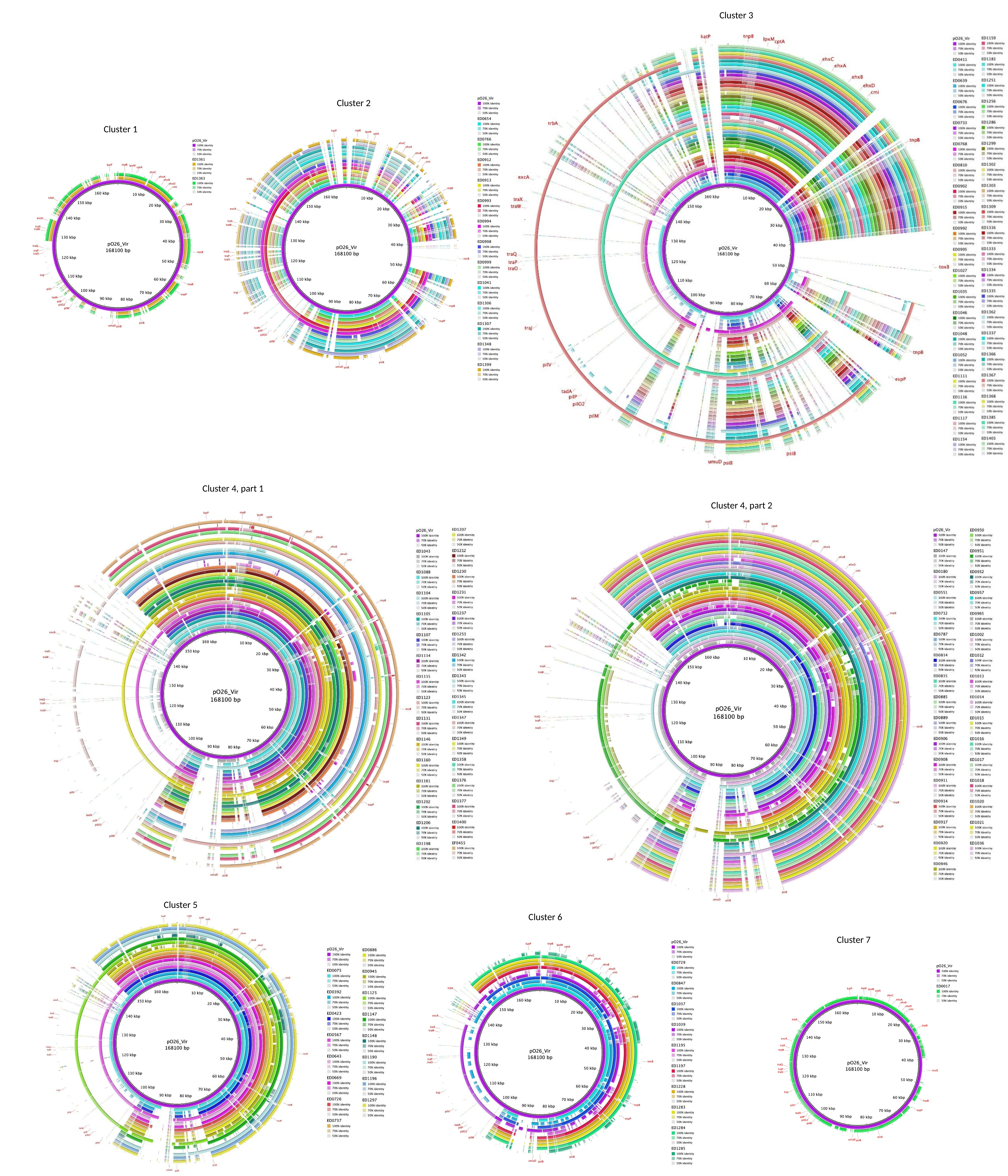

Cluster 3
Cluster 2
Cluster 1
Cluster 4, part 1
Cluster 4, part 2
Cluster 5
Cluster 6
Cluster 7

Supplement: Supplementary file 3 [file Presentation_1.pptx]

## Slide 1
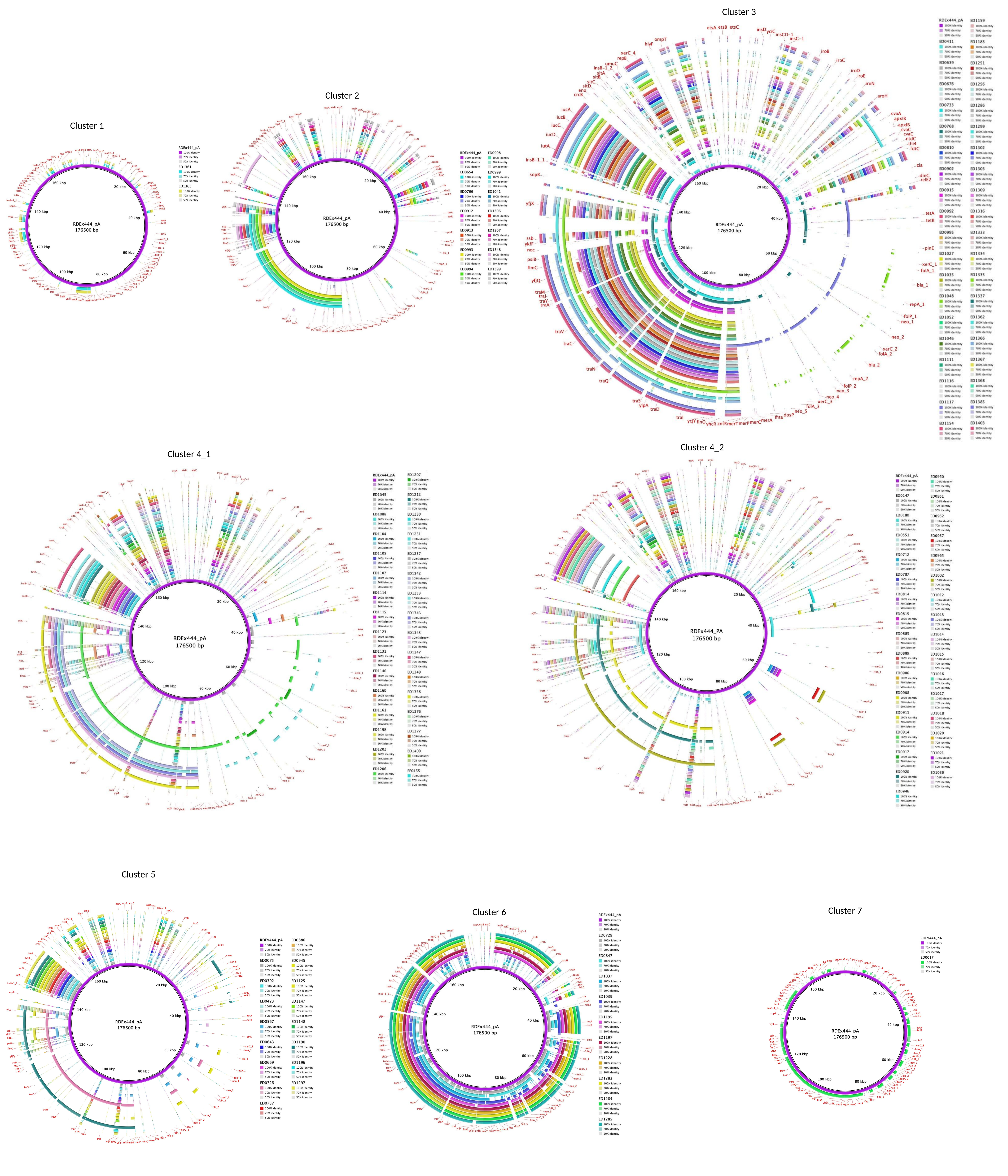

Cluster 3
Cluster 2
Cluster 1
Cluster 4_2
Cluster 4_1
Cluster 5
Cluster 7
Cluster 6

Supplement: Supplementary file 4 [file Presentation_2.pptx]
